# Penny Wisdom Again

**Published:** 1886-08

**Authors:** 


### PENNY WISDOM AGAIN—SAVE AT THE SPIGOT, LOSE AT THE BUNG.

Wm. Wood & Co., heretofore so liberal, have done a shabby thing which not only lowers them in the estimation of Southern and Western journals, but which will cause them the loss of many a dollar. We cannot account for it, unless they are feeling the falling off of Southern and Western patronage in consequence of the silly and suicidal policy they allowed the manager of the *Record* to pursue with reference to the International Congress. It will be remembered the *N. Y. Medical Record* (Wood & Co.'s leading publication) was very bitter agaist the South and West, really insulting; and it is not a matter of surprise that many of the subscribers of the *Record* have dropped it.

Wood & Co. have notified (we presume all of the) Southern and Western medical journals that hereafter books sent for review will be sent by express at the expense of those receiving them.

We would like to ask, who is most benefitted in a transaction of that kind? Are not reviews advertisements? Intended as such, to promote the sale of the books? Well, publishers have been giving such notices, generally commendatory, in consideration of a complimentary copy. Where the editor gets three dollars worth of books, Wood & Co. receive perhaps twenty-five dollars worth of advertising, to say nothing of the fruits of it in the way of sales; and now they want the journals of the South and West to pay the express charges. None of it for us, if you please.

It has been proposed that the journals of the South and West unite in declining to receive any more books, and give any more reviews on any such terms. The *St. Louis Medical Review* and the *St. Louis Medical and Surgical Journal* are with us in this move, and at the risk of being accused of a boycott, we ask others to join us. Other publishing houses, the great Appletons and Blakistons make no such unreasonable demands, and the journals of this section of the country should resent any such proposition, as niggardly and unworthy of a live, and heretofore liberal publishing firm.

